# Interference between smooth pursuit and color working memory

**DOI:** 10.16910/jemr.10.3.6

**Published:** 2017-07-10

**Authors:** Shulin Yue, Zhenlan Jin, Fan Chenggui, Zhang Qian, Ling Li

**Affiliations:** 1 University of Electronic Science and Technology of China , Chengdu , China; 2 Key Laboratory for NeuroInformation of Ministry of Education, University of Electronic Science and Technology of China , Chengdu , China

**Keywords:** color WM, smooth pursuit, spatial attention, retention period, delayed-matchto-sample paradigm, dual-task

## Abstract

Spatial working memory (WM) and spatial attention are closely related, but the relationship between non-spatial WM and spatial attention still remains unclear. The present study aimed to investigate the interaction between color WM and smooth pursuit eye movements. A modified delayed-match-to-sample paradigm (DMS) was applied with 2 or 4 items presented in each visual field. Subjects memorized the colors of items in the cued visual field and smoothly moved eyes towards or away from memorized items during retention interval despite that the colored items were no longer visible. The WM performance decreased with higher load in general. More importantly, the WM performance was better when subjects pursued towards rather than away from the cued visual field. Meanwhile, the pursuit gain decreased with higher load and demonstrated a higher result when pursuing away from the cued visual field. These results indicated that spatial attention, guiding attention to the memorized items, benefits color WM. Therefore, we propose that a competition for attention resources exists between color WM and smooth pursuit eye movements.

## Introduction

Working memory (WM) (Baddeley, 2000; Baddeley & Hitch, 1974; Engle, 2002)facilitates temporary maintenance of relevant information in the mind and plays a critical role in many complex cognitive tasks. Visual WM stores and manipulates visual and spatial information and these information are stored relatively separate within the visuospatial sketchpad (Baddeley, 2000) . Spatial WM and spatial attention are closely related (Awh & Jonides, 2001; Cowan, 2000) and spatial attention plays an important role in maintaining location information (Awh & Jonides, 2001) .

As widely accepted, spatial attention and eye movements are intimately coupled (Posner, 1980; Reeves & Sperling, 1986; Kurylo, Reeves, & Scharf, 1996; Kowler, Anderson, Dosher & Blaser, 1995; Kustov & Robinson, 1996; Moore & Fallah, 2004) . Shift of spatial attention can be overt or covert and eye movements demonstrate overt shift of spatial attention. Therefore, it is natural that eye movements interact with spatial WM. Refixations on an object when freely inspecting a scene improve the memory performance and it was proposed that refixations continuously update the availability of items in the memory (Zelinsky & Loschky, 2009) . On the contrary, there are also studies demonstrating the interference of eye movements on the spatial WM (Baddeley & Lieberman, 1980; Kerzel & Ziegler, 2005; Smyth & Scholey, 1994) . In line with the close relationship between spatial WM and attention, Lawrence, Myerson, and Abrams (2004 ) found that the spatial WM span was decreased by the covert shift of attention and even more by eye movements, demonstrating that eye movements interfere with spatial WM and the interference caused by eye movements is greater than covert attention shift. Moreover, keeping a location in memory curved eye trajectories away from the remembered location (Theeuwes, Olivers, & Chizk, 2005) . These studies demonstrate an intimate link between the control of eye movements, attention and working memory (Golomb, Chun, & Mazer, 2008; Theeuwes, Belopolsky, & Olivers, 2009).

In contrast with the clear relationship between spatial WM and eye movements, the relationship between eye movements and non-spatial WM is still under debate. In the study of Awh, Jonides, and Reuter-Lorenz (1998 ), a letter appeared and subjects had to memorize the location of the letter or the identity of the letter. At the same time, choice stimuli appeared during the retention interval and subjects indicated the shape of the stimuli by pressing button quickly. Reaction times to choice stimuli were faster if they appeared at the memorized location, suggesting that spatial attention is focused on the memorized location. In contrast, when remembering the identity of the letter, no benefit of the location of the letter was observed, suggesting the specific relation between spatial WM and visual attention. Similarly, in the study of Lawrence, Myerson, and Abrams (2004 ), the authors found a reduction in the spatial WM span by the attention shift, but not in the verbal WM. In line with these findings, a movement discrimination task interferes with the spatial WM task of memorizing dot locations, while a color discrimination task does not (Klauer & Zhao, 2004) . Additionally, Kerzel and Ziegler (2005) found that the color WM performance was unaffected by the addition of smooth pursuit task while the spatial WM performance was impaired by the additional smooth pursuit task. On the contrary, Makovski and Jiang (2009) found concurrent performance of a color WM task and attentive tracking task produced mutual interference with each other. In their study, subjects were encouraged to maintain fixation at the display center while attentively tracking multiple objects. They proposed that common attentional processes are engaged in these two tasks which are of central and amodal origin. Baddeley (2000) proposed a multiple component model, which included central executive, visuospatial sketchpad, phonological loop and episodic buffer. Furthermore, visual and spatial information were stored relatively separately within the visuospatial sketchpad. Therefore, we assumed that the common attentional resources shared by attentive tracking and color WM point to the central executive component in Baddeley’s model (2000).

Similar to the intimate couple of visual spatial attention and saccades, smooth pursuit eye movements foveate moving objects and are closely related to visual attention as well. Dividing attention from the smooth pursuit could impair the pursuit performance (Acker & Toone, 1978; Brezinova & Kendell, 1977; Heinen, Jin, & Watamaniuk, 2011; Souto & Kerzel, 2008) . Addition of larger random dot cinematogram (RDC) that moves with the pursuit target helps to release attention from the pursuit which can be used to improve performance of secondary attention task (Jin et al., 2013) . These results demonstrate that attention is involved in the smooth pursuit eye movements. In addition, studies showed that attention is narrowly allocated around the smooth pursuit target although with some conflicts. Some studies showed that attention allocation during smooth pursuit is asymmetric, more attention is distributed ahead of the pursuit target (Kanai, van der Geest, & Frens, 2003; Khan, Lefe`vre, Heinen, & Blohm, 2010; Seya & Mori, 2012; Smeets & Bekkering, 2000; van Donkelaar & Drew, 2002) , while others proposed that attention is symmetrically distributed around the pursuit target (Lovejoy et al., 2009; Watamaniuk & Heinen, 2015) . Studies on schizophrenia demonstrated that spatial WM impairment was associated with dysfunctions in the oculomotor mechanisms (Park & Holzman, 1993; Snitz et al, 1999) . The deficit of spatial WM performance was related to smooth pursuit because of the limitation of attention distributed to position (Kerzel & Ziegler, 2005; Park, Holzman, & Levy, 1993) . However, color WM performance was unaffected by the addition of smooth pursuit task, suggesting the allocation of attention was restricted to position which is responserelevant dimension (Kerzel & Ziegler, 2005) . These studies again implied the ambiguity of the relationship between eye movements and non-spatial WM.

Therefore, we designed a delayed-match-to-sample paradigm (DMS), whereby a WM load task is interleaved with pursuit task during the maintenance period in the present study. In the experiment, subjects memorized the color of squares in the cued visual field and foveated a moving cross during the retention period. The cross moved toward or away from the visual field where the memory targets were presented. Since attention is narrowly distributed around the pursuit target, pursuing towards or away from the cued visual field might influence the color WM differently because the attention would be allocated in the cued VF or in the uncued VF in the present design. Therefore, we asked whether eye movements affect the performance of color WM and whether smoothly directing gaze toward or away from the previous location of the memory item would have differential effects on the color WM.

## Methods

### Participants

Sixteen college students (five females and eleven males, mean age 22.8) with normal vison participated in the experiment. All subjects had no history of neurological diseases and were completely naïve to the aim of the current study. The study was approved by the University of Electronic Science and Technology of China Ethics Board and the methods were performed in accordance with the approved guidelines and all experiments conformed to the declaration of Helsinki. All subjects signed written consent form before participating in the study.

### Apparatus and visual stimuli

The stimuli were generated by Psychtoolbox (Pelli, 1997) in MATLAB and were presented on a 1024×768 pixels display with a refresh rate of 60 Hz. Eye movement data were recorded by EyeLink1000plus eye-tracker (SR Research Ltd., Kanata, Canada) with sampling rate of 2000 Hz. The tracker was calibrated and validated through the way in which the observer fixated nine locations distributed across the display using a standard software routine provided with the EyeLink system. Subjects were seated on a chair in darkness with their head stabilized using a chin and forehead rest and viewed the stimuli display from a distance of 65cm.

Colors of the WM stimuli were made by adobe fireworks with uniform luminance, saturation and resolution. There were nine colors to be picked. Each colored square was 0.88 degree in width and could be presented at one of four potential locations in each visual field. The colored squares were arranged along imaginary lines which were displaced 3.86 degree to the left and right of fixation and the vertical distance between squares was 0.88 degree.

### Procedure

**Figure 1:**
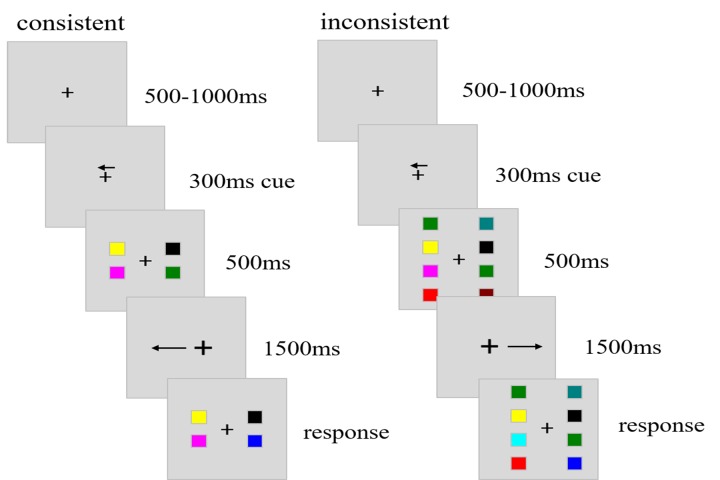
Schematic representation of stimuli sequence in the WM + pursuit session. The cue pointed to the left and right before the onset of the target stimuli, which consisted of 2 or 4 colored squares in both VFs. Subjects were required to memorize the color of the squares in the cued VF and judged whether the colors of the test stimuli in the cued VF were same or not. During the retention period, subjects pursued the cross which moved towards or away from the cued VF. According to the direction of the cross motion, the trials were grouped into consistent and inconsistent trials.

The study included three sessions, the WM + pursuit session, the WM-only session and pursuit-only session. The WM-only session and pursuit-only session is basically same as the WM + pursuit session, so we will describe this session as a representative in details below. Each trial began with a random duration (500-1000 ms) of central cross (0.49 degree in length), which was followed by a 300 ms cue (0.49 degree in length) pointing to left or right with equal probability. The WM target stimuli appeared after the cue and stayed on the display for 500 ms. The target stimuli consisted of 2 or 4 colored squares in both left and right visual fields (VFs) in addition to the central cross Figure 1. In trials with 4 squares (load 4 condition), the squares were presented at all four possible locations in each visual field. Meanwhile, in trials with 2 squares (load 2 condition), the squares were always presented at two locations near the horizontal meridian in each visual field. These constant positions of squares in each condition were aimed to diminish the potential effect of location change across trials. At the same time of the target stimuli offset, the fixation cross jumped back 0.88 degree and moved for 1500 ms towards the left or the right visual field at the constant velocity of 5.26 deg/s. The backward 0.88 degree step contributed to reduce the occurrence of catch-up saccades (Rashbass, 1961). The motion direction of the cross was consistent or inconsistent with the cue direction and each type occupied 50% of trials. This manipulation generated four types of trials, each type occupied a quarter of trials. After the moving cross disappeared, the test stimuli were presented and stayed on the display until the response. Colors of the squares in the cued VF of the test stimuli were either same as those in the cued VF of the target stimuli or one of them changed its color. Similarly, the colors in the uncued VF of the test stimuli were same as those in the uncued VF of the target stimuli or one of them changed its color with equal chance. The change of square colors in the cued and uncued VF were independent each other. As soon as the test stimuli appeared, subjects were instructed to click the left (Yes) or right (No) button of the mouse quickly to indicate whether the colors in the cued VF changed or not with maximum time window of 3 seconds.

The WM-only session was same as the WM + pursuit session except that the central cross was static and stayed at the display center during the retention period. Subjects conducted the WM task as in the WM + pursuit session and fixated at the central cross during the retention period. In the pursuit-only session, both the target and test stimuli for the WM task were not presented during the target presentation period and test stimuli period, but only the central cross.

Subjects practiced the pursuit task until they could pursue smoothly before collecting the data. Each subject completed 4 blocks of the main experiment (WM and pursuit dual-task) and 2 blocks of each control experiment. All blocks in the study had 64 trials, so each subject produced 512 trials in total.

### Data analysis

Eye movement data were analyzed offline. Horizontal and vertical eye velocities were calculated offline from the recorded position signals by differentiating and filtering (2-pole Butterworth noncausal filter, cutoff = 50 Hz). Saccade detection used an empirically-chosen threshold of 25°/s. For the pursuit data analysis, the open-loop period was considered to have a duration of 150 ms, with an onset of 150 ms after the stimuli motion onset (average pursuit latency over all subjects was 152 ms). Openloop gain was computed by dividing average eye velocity over a 20 ms bin centered 300 ms after the stimuli motion onset (the end of open-loop period) by stimuli velocity. Steady-state gain was computed by dividing mean eye velocity in a 450-950 ms time window by stimuli velocity.

Trials with failure of eye position recording or with blink which occurred from the cue onset to the end of motion, were excluded from the analysis. In addition, trials with eyes deviating from the fixation more than 2o during the last 100 ms of the target stimuli presentation were discarded as well to assure the starting position of the pursuit. After these trial removals, 88.82% of trials survived on average in all subjects.

## Results

**Figure 2:**
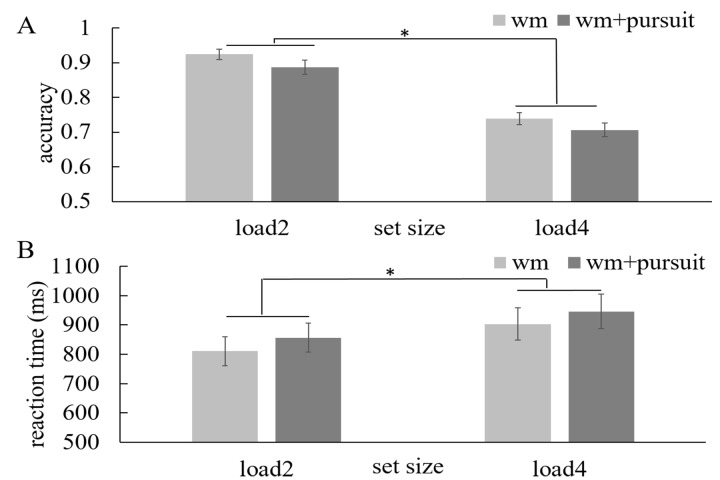
The WM performance with and without pursuit task. Light grey indicates the WM performance in the WM-only session and dark grey indicates the WM performance in the WM + pursuit session. (A) accuracy, (B) reaction times. Error bars represent standard error and asterisks indicate significance at p < 0.05.

First, we compared the WM performance in the WM + pursuit session and the WM-only session to investigate whether adding secondary pursuit task in the retention period would harm the WM performance. To do this, we have conducted a two-way repeated-measure ANOVA using the WM load (load 2 and 4) and the presence of pursuit task (without and with pursuit task) as factors Figure 2. As usual, we found the WM accuracy decreased with higher load (F(1,15)=270.97, p=0.000) and adding the pursuit task did reduce the WM accuracy (F(1,15)=9.27, p=0.008). Consistently, the reaction times were longer with higher load (F(1,15)=11.94, p=0.004) and adding the pursuit task lengthened the reaction times (F(1,15)=4.83, p=0.044) as well. These results demonstrated that eye movements during the retention period harmed the WM performance.

We also compared the pursuit performance in the WM + pursuit session and the pursuit-only session to investigate whether adding secondary WM task would affect the pursuit performance. We found a significantly lower peak open loop gain (F(1,15)=14.39, p=0.002) in the WM + pursuit session (0.77 ± 0.05) compared with pursuit-only session (0.89 ± 0.05). In addition, the steadystate velocity gain was also lower in WM + pursuit session (0.92 ± 0.024) than in the pursuit-only session (0.98±0.014) (F(1,15)=10.57, p=0.005). These results indicated the addition of the secondary WM task harmed the pursuit performance.

Combining these results, we see that the WM and pursuit interfere with each other. However, it is still unclear whether it is general for secondary task or specific to the eye movement type. To check this, we analyzed the data from the WM + pursuit session in details.

**Figure 3:**
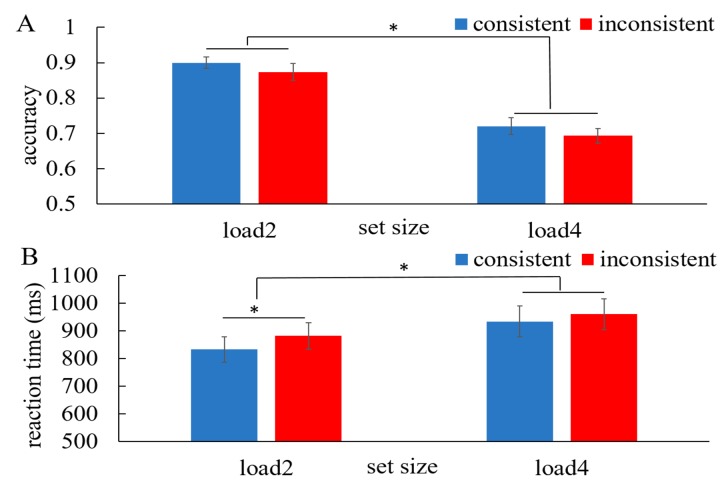
The performance of color WM in the WM + pursuit session. (A) accuracy. (B) reaction time. Error bars represent standard error. Asterisks indicate significance at p < 0.05.

Figure 3 shows the performance of working memory in the WM + pursuit session, which was assessed using a two-way repeated-measure ANOVA for load (load 2 vs. load 4) and consistency (consistent vs. inconsistent). Consistent with previous findings, the accuracy was decreased with higher load (F(1,15)=92.53, p=0.000) and the consistent condition revealed higher accuracy than the inconsistent condition (F(1,15)=4.73, p=0.046). No interaction between the load and consistency. Similarly, the reaction times increased with higher load (F(1,15)=14.50, p=0.002) and the consistent condition showed shorter reaction times than the inconsistent condition (F(1,15)=5.23, p=0.037). Further post hoc t-tests, Bonferroni corrected for multiple comparisons, showed longer reaction times in the inconsistent condition (881.6 ± 48.1 ms) than in the consistent condition (832.3 ± 45.6 ms) (Bonferroni adjusted t(15) = 3.15, p = 0.007) with load 2. In this comparison, the significance level was adjusted to 0.0083 (0.05/6 = 0.0083) due to 6 possible comparisons across the 4 conditions (4*3/2 = 6).

Combining the accuracy and reaction time results, we found pursuing towards the locations where the WM stimuli were presented produced better WM performance than pursuing away from those during the retention period. In addition, we computed the mean horizontal eye positions in a 200 ms time window since the test display onset to see whether eyes returned to the central fixation cross. A two-way repeated-measures ANOVA on load (load 2 vs. load 4) and consistency (consistent vs. inconsistent) showed a significant effect of consistency (F(1,15)=5347.64, P=0.000) and interaction between load and consistency (F(1,15)=14.46, P=0.002). Further comparison showed that the eyes deviated more from the fixation cross with load 2 (9.857 degree) compared with load 4 (9.510 degree) (t(15) = 2.59, p = 0.021). These data showed that the eyes posited around the positions where pursuit ended and had not returned to the fixation cross yet when the test display appeared, resulting in bigger retinal eccentricity in the inconsistent condition. Interestingly, the retinal eccentricity was smaller with load 4 than with load 2, indicating that memorizing more colors urged eye to return to the central fixation.

**Figure 4:**
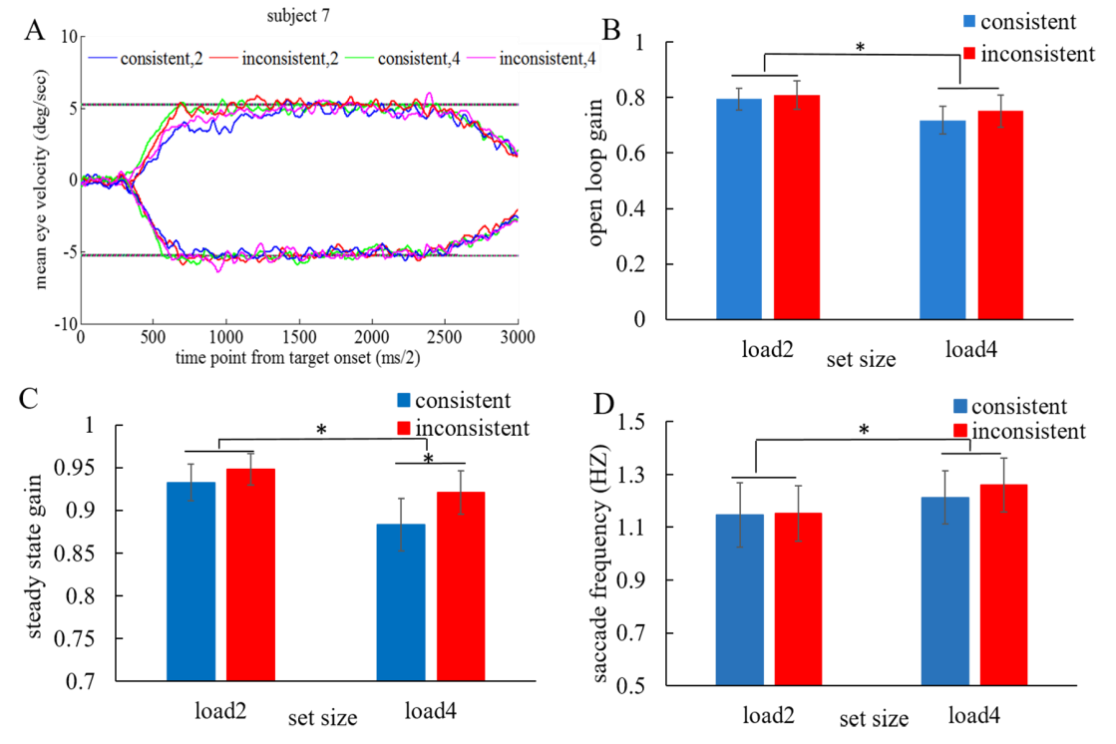
The pursuit performance in the WM + pursuit session. (A) Mean eye velocity trace in all conditions from target onset for a representative subject. The two horizontal line means the target velocity. (B) Peak open loop acceleration for all subjects averaged in the open loop period. (C) Eye velocity gain for all subjects averaged in the steady state. (D) Saccade frequency in the retention interval. Error bars represent standard error. Asterisks indicate significance at p < 0.05.

In addition, to check the effect of the WM on the pursuit, we analyzed the open-loop gain and steady-state gain using a two-way repeated-measures ANOVA using load (load 2 vs. load 4) and consistency (consistent vs. inconsistent) as factors respectively Figure 4. The ANOVA revealed lower open-loop gain (F(1,15)=29.50, p=0.000) and steady-state gain (F(1,15)=10.08, p=0.006) with higher load. Moreover, the steady-state gain was higher in the inconsistent condition (F(1,15)=8.30, p=0.011) and there was a marginally significant interaction between the load and consistency (F(1,15)=3.85, p=0.069). Further pairwise comparison using 0.0083 (0.05/6 = 0.0083) as the significance level due to 6 possible comparisons across the 4 conditions (4*3/2 = 6) showed mean steady state gain in the inconsistent condition was higher than that in the consistent condition with higher WM load (Bonferroni adjusted t(15) = 3.28, p=0.008). Additionally, we also found higher saccade frequency with higher load (F(1,15)=9.31, p=0.008), indicating the worse pursuit performance with higher load. Overall, these results showed that the pursuit performance was further impaired by higher load and the steady-state performance was even influenced by the consistency between the WM and the pursuit.

In order to check whether the change of squares’ colors in the uncued VF affected the WM performance, we grouped the trials in two types in each load condition for the data from the WM only session. One type included trials with the color change (change) and the other type included trials without color change (same) in the uncued VF. Since the color changes in the cued and uncued VF were independent each other and all trial type had equal opportunities, we would not expect an effect of the trial type. We assessed the WM performance using the repeated-measures ANOVA for WM load (load 2 vs. load 4) and trial type (same vs. change). There was only a significant effect of load for accuracy (F(1,15)=300.95, p=0.000) and no effect of the trial type was found, indicating that the color change in the uncued VF did not affect he WM performances. Therefore, we suggested that the subjects followed the instruction.

## Discussion

In the current study, we showed that the color WM and the smooth pursuit eye movements interfered with each other. More interestingly, pursuing towards the cued visual field resulted in enhanced color WM performance compared with pursuing away from it. In turn, the higher load of WM impaired the pursuit performance more than the lower load of WM.

The color WM performance was impaired by adding the pursuit task during the retention period and the pursuit performance was also impaired by adding the color WM task, demonstrating that the color WM and smooth pursuit interfere each other and further suggesting smooth pursuit share common resources. We think that common attentional resources shared by the color WM and SPEM might be of central, amodal origin, which might point to the central executive component in Baddeley’s model (2000). This also agrees with the resource model of working memory which propose that a limited resource is distributed flexibly across all representations which are maintained in memory (Ma, Husain & Bays, 2014) . Consistently, higher WM load impaired the pursuit performance more as shown by the lower open-loop gain and steady state gain, and higher saccade frequency in the load 4 condition.

In turn, greater impairment of the color WM performance which was shown by lower accuracy and longer reaction time, was observed in the inconsistent condition where the pursuit during the retention period was away from the location of the WM stimuli, showing that the direction of smooth pursuit plays a role in our WM task. In the current study, the fixation cross moved towards or away from the cued VF from the display center. Therefore, in the trials where the cross pursued towards the cued VF, the smooth pursuit target moved within the cued VF during the whole retention period and vice versa. Studies agreed that attention is narrowly distributed around the pursuit target, while they disagree about the symmetry of the attention distribution during smooth pursuit (Kanai, van der Geest, & Frens, 2003; Khan, Lefe`vre, Heinen, & Blohm, 2010; Seya & Mori, 2012; Smeets & Bekkering, 2000; van Donkelaar & Drew, 2002; Lovejoy et al., 2009; Watamaniuk & Heinen, 2015) . Hence, we propose that more attention could be distributed in the cued VF during the retention period in the consistent condition and more attention benefited the color WM. The results showed that shifting attention smoothly to the locations where the memory target was presented would help the color WM compared to when shifting attention away from it. These results mimic attention-based rehearsal hypothesis (Awh & Jonides, 2001) . This hypothesis proposes the rehearsal of stored spatial information is accomplished by shifts of spatial selective attention to memorized locations. Therefore, our finding further extends the hypothesis to the relationship between spatial attention and non-spatial WM and further suggest that spatial attention plays a functional role in maintaining non-spatial information. However, our results could not exclude the possible effect caused by the closer eye position to the WM target in the consistent condition at the onset of the test display.

At the same time, the pursuit performance was better in the inconsistent condition than in the consistent condition during the steady-state, especially when the WM load was higher. Since spatial attention plays an important role in maintaining location information (Awh & Jonides, 2001) and attention is allocated around the pursuit target, a conflict or competition of attention resources might occur in the inconsistent condition, especially when the demand for attentional resources is intensified. This imitates the anti-saccade task, which requires subjects to suppress a reflexive saccade towards a visual stimulus and perform a voluntary saccade away from the target (Cutsuridis et al. 2007; Everling & Fischer 1998) . Therefore, the pursuit in the inconsistent condition was more difficult than that in the consistent condition and more cognitive control is needed for the pursuit in the inconsistent condition. This is in line with the previous finding that eye-target synchronization improved under higher cognitive load (five-words) for normal subjects (Contreras et al., 2011) .

Taken together, our results are in line with the finding from Makovski and Jiang (2009) and support that the visual WM and smooth pursuit share common attentional resources. Due to the presentation of the colored squares on the display, it is still possible that the locations of squares were encoded and memorized automatically. However, we tried to reduce this possibility by fixing the possible locations of the squares in each condition and forbidding exchanging colors between squares when comparing the colors of the target and test stimuli. Such a design reduced possible effect of stimuli location, so we suggest that our results can be interpreted as the relationship between the color WM and smooth pursuit. Many studies have shown persistent neural activities during the retention period of WM in the prefrontal areas (Funahashi et al., 1993) , frontal eye fields (Curtis et al., 2004; Tark & Curtis, 2009) and posterior parietal cortex (Schluppeck et al., 2006). Since smooth pursuit-related brains areas mainly involve fronto-parietal network, such as the frontal eye field, supplementary eye fields and posterior parietal cortex, there are many overlaps in the brain areas related to the WM and smooth pursuit. Similarly, we supposed that there might be a competition for the shared neural resources of fronto-parietal network, resulting in competition between the color WM and pursuit tasks.

In sum, the current study found mutual interference between the color WM task and smooth pursuit eye movements. Furthermore, the pursuit direction plays a role in the color WM that the color WM benefited when pursuing towards the locations where the WM stimuli were presented. We propose that it is because more attentional resources are directed to the locations of the WM stimuli during the retention period by the help of the smooth pursuit eye movements.

### Ethics and Conflict of Interest

The authors declare that the contents of the article are in agreement with the ethics described inhttp://biblio.unibe.ch/portale/elibrary/BOP/jemr/ethics.html and that there is no conflict of interest regarding the publication of this paper.

### Acknowledgements

This work was supported by grants from Natural Science Foundation of China (Nos 61673087, 61473062 and 61203363), and the Fundamental Research Funds for the Central Universities.

S.Y. conducted the experiment, analyzed data and wrote the manuscript. Z.J. designed the experiment and revised the manuscript. C.F. prepared figures and Q.Z. proofed article. L.L. designed the experiment. All authors reviewed the article.
